# The role of depth of encoding in attentional capture

**DOI:** 10.3758/s13423-015-0807-6

**Published:** 2015-02-18

**Authors:** Edyta Sasin, Mark Nieuwenstein, Addie Johnson

**Affiliations:** Department of Experimental Psychology, University of Groningen, Grote Kruisstraat 2/1, 9712 TS Groningen, The Netherlands

**Keywords:** Attention, Working memory, Attentional capture, Depth of encoding

## Abstract

The aim of the current study was to examine whether depth of encoding influences attentional capture by recently attended objects. In Experiment 1, participants first had to judge whether a word referred to a living or a nonliving thing (deep encoding condition) or whether the word was written in lower- or uppercase (shallow encoding condition), and they then had to identify a digit displayed midway in a rapid serial visual presentation (RSVP) stream of 8 pictures. A picture corresponding to the previously processed word was presented either before or after the target digit. The results showed that this picture captured attention, thus resulting in an attentional blink for identification of a target digit, in the deep encoding condition but not in the shallow encoding condition. In Experiment 2, this capture effect was found to be abolished when an additional working-memory (WM) task was performed directly after the word-judgment task, suggesting that the capture effect stemmed from residual WM activation that could be erased by means of a secondary WM task. Taken together, these results suggest that deep and shallow encoding result in different degrees of WM activation, which in turn influences the likelihood of memory-driven attentional capture.

Working memory (WM) is needed to maintain task goals and the information relevant to achieving these goals (Baddeley, 2010; Carlisle et al. 2011; Cowan, 2005). The limited capacity of WM requires that pertinent information be attended, and that irrelevant information be inhibited (e.g., Cowan, 2010). Given the role of WM in the control of intentional acts, it is not surprising that attention and WM are strongly intertwined (Awh & Jonides, 2001; Awh et al. 2006). Indeed, there is evidence from visual search studies that it can be hard to ignore distractor objects when they match representations held in WM (e.g., Downing, 2000). For example, Soto and colleagues (Soto et al. 2005; Soto et al. 2006) found that reaction times in a search task were slower when one of the distractors in the search array shared a feature with an item that was retained in working memory, indicating that attention can be captured by items that match an item held in memory, despite its irrelevance to current task goals.

The finding that information held in WM can influence visual selection is consistent with Pillsbury’s (1908) hypothesis that searching for anything requires forming a mental image of the target. Pashler and Shiu (1999) tested whether imagining an item would trigger search even when such search impairs performance on a current task. In this study, participants were asked to identify a digit target that was embedded in an RSVP sequence of pictures and that could be preceded or followed by a picture of a previously imagined object. The results showed that digit identification was impaired when this picture was shown before the digit target, suggesting that the picture captured attention, thereby causing an attentional blink (i.e., less accurate detection of a target when it follows within less than 500 ms from a preceding target or capture stimulus; Raymond et al. 1992; see also, Nieuwenstein et al. 2009). Pashler and Shiu’s results can therefore be taken as support for Pillsbury’s hypothesis that forming a mental image is sufficient to instigate a bias towards attending that object when it is subsequently encountered again. Importantly, Pashler and Shiu also found that attention was still directed to a picture of a previously imagined object even when the instructions encouraged participants to discard the mental image. To the extent that this instruction could lead participants to intentionally discard and thus forget the imagined object (see, e.g., Vogel et al. 2005), this finding suggests that the recent activation of an item may be sufficient to elicit the attentional capture effect. Therefore, one explanation for Pashler and Shiu’s (1999) finding of attentional capture by an imagined object is that the effect is driven by residual WM activation of imagined object that persisted through the trial, despite the instruction to discard the image. Pashler and Shiu’s results may be then interpreted as support for the hypothesis that items that match the content of WM guide visual attention.^1^

Although many studies have investigated the conditions under which the maintenance of an object in working memory does and does not result in attentional capture (e.g., Olivers et al. 2011), only little is known about the influence of previous processing on attentional capture. In the current study, we addressed this matter by investigating whether depth of encoding influences attentional capture. According to the levels of processing framework (Craik & Lockhart, 1972), incoming stimuli can be analyzed to a different depth of encoding ranging from shallow, sensory analysis based on structural aspects of the word to deeper, semantic analysis involving the retrieval of meaning and implications, and the deeper the processing, the better is memory for the processed words (Craik & Tulving, 1975) as participants have been found to remember more words if a task requires the analysis of word meaning than if the task only requires an analysis of the structural aspects of a written word.

The current study examined whether depth of encoding influences the deployment of selective attention towards previously processed items. Our hypothesis was that the deeper the encoding of a word prior to an RSVP trial, the more likely it would be that a picture depicting the referent of this word would capture attention in the RSVP stream, even though attending to the picture would impair target identification.

## Experiment 1

Experiment 1 addressed whether depth of encoding impacts the likelihood of attentional capture by a previously processed word. This was done by comparing the attentional capture effects for pictures that matched a word that had either undergone deep or shallow encoding. In the deep-encoding condition, participants made an animacy judgment for a word, whereas the shallow-encoding condition required participants to judge whether a word was printed in upper-or lowercase. To determine whether the manipulation of depth of encoding was effective, we included an incidental memory test at the end of study, which aimed to determine whether participants could remember the previously processed words better if they had undergone deep as opposed to shallow encoding.

### Method

#### Participants

Forty-three students, enrolled in the English language psychology bachelor program at the University of Groningen, participated in the experiment for partial course credit, and an additional 16 volunteers from the University of Groningen community were paid in exchange for their participation. All participants had normal or corrected to normal visual acuity (42 females; *M* = 22.9 years, *SD* = 5.65). The study was approved by the ethics committee of the psychology department. Informed written consent was obtained.

#### Apparatus and stimuli

Stimulus presentation and response collection were controlled by a program written with E-Prime 2.0 (Schneider et al. 2002) and the experiment was done on computers that were fitted with 22-inch CRT computer monitors with a refresh rate of 100 Hz and a resolution of 1280 × 1024 pixels.

The word stimuli used in the experiment were 120 high-frequency English nouns of high imageability, 479–655 (*M* = 593.88), according to the Paivio (Paivio et al. 1968) norms. The English Lexicon Project (ELP) database (Balota et al., 2007) was used to select words of high frequency according to the Hyperspace Analogue to Language (HAL) frequency norms (Lund & Burgess, 1996). We selected words that had a frequency of 20 per million or greater (Brysbaert & New, 2009) and that were familiar to the participants in our study. All words were displayed in Courier New, 20-point font. The picture stimuli were line drawings taken from the Snodgrass and Vanderwart (1980) and International Picture Naming Project (IPNP) sets (Szekely et al., 2005). Drawings of 209 nouns were used as stimuli in the experiment (129 as fillers and 80 as targets or fillers in the RSVP stream), and each measured approximately 9.5 $\times$ 9.5 cm (7.76° of visual angle). Word stimuli presented in the experimental task were composed of the names of the 80 target pictures. Words were generated from the pool of 40 words used in the animacy-judgment task (20 living and 20 nonliving) and 40 words used in the case-judgment task (20 uppercase and 20 lowercase). Each subset of words used in the case-judgment task contained 10 living and 10 nonliving words. An additional 32 words were selected for a practice blocks. The digits 2–9 were used as target digits and were presented in 40-point Courier New font (measuring 0.42° of visual angle horizontally and 0.82° vertically). The pattern mask following the digit was composed of randomly distributed black lines and measured 2 $\times$ 2 cm (1.62° of visual angle). An additional 36 pictures were selected for a practice block. All stimuli were displayed in black on a white background at the center of the screen.

#### Procedure

The experimental task was preceded by a practice phase in which the word and digit tasks were practiced in isolation. In the 32-trial digit practice phase, participants viewed RSVP streams of eight pictures (presented for 120 ms each) with a masked target digit shown after the fourth picture in the sequence. The participant’s task was to identify the target digit by typing it in after the presentation of the RSVP stream. Feedback was given after each response by displaying the correct response. The duration of the digit was set to 110 ms initially, and the duration of the mask was set to 20 ms. During the practice trials, whenever the correct answer was given three times in a row, the digit presentation time was reduced by 20 ms and the duration of the mask was increased by 20 ms. If the next three trials again each yielded a correct answer, the presentation time of the digit was again reduced, but now by 10 ms and the mask duration increased by 10 ms. Whenever an incorrect answer was given three times in a row, the duration of the digit was likewise increased by 20 or 10 ms (and the mask duration decreased by 20 or 10 ms, respectively). The target duration at the end of the practice trials was used as the initial duration for digit presentation during the experimental trials. The word-practice phase included separate blocks for the animacy and case-judgment tasks. One block of 16 trials of each task was completed, with the order of the blocks counterbalanced across subjects. Words were presented one at a time and participants had to make an animacy or case judgment as accurately and as quickly as possible. After each response, feedback was provided indicating whether the response was correct or incorrect.

In the experimental phase, subjects performed both the word-judgment and digit-identification tasks on each trial. The experimental phase was blocked, with approximately half the participants performing the animacy-judgment task first and the other half performing the case-judgment task first. Participants were asked to focus only on reporting the digit after having completed the word task. The words used for the animacy- and case-judgment tasks were selected at random from the set of available words.

At the beginning of each trial, the word to be judged appeared on the screen. Participants responded by pressing the “c” key for words that referred to living beings or words printed in lowercase and the “m” key for nonliving things or words printed in uppercase. Next, there was a 500-ms fixation period. Ten objects were then presented in an RSVP stream: four pictures, the target digit and its mask, and another four pictures. The *critical* picture (the picture corresponding to the word seen at the beginning of the trial) was presented in position 3 or 7, and the digit and its mask occupied positions 5 of the RSVP sequence. After the sequence finished, participants responded by typing in the digit they identified. The correct digit response was then provided as feedback. During the experimental trials, the duration of the target digit and its mask was adjusted using a similar method as that used during practice, with the difference being that target duration was decreased when the accuracy fell below 70% or increased when accuracy was above 90% in the last 10 trials. The minimum target duration was 20 ms.

After the experimental phase was finished, there was an unexpected recognition test that required participants to indicate whether a word had been shown earlier on in the experiment. During the recognition test, 40 words from the animacy-judgment task and 40 words from the case-judgment task were shown randomly intermixed with 40 words which had not been presented during the experiment. Words were shown one at a time, and each word remained in view until participants indicated whether they had seen it before in the experiment. The recognition judgment had to be made as accurately and as quickly as possible. Once the experiment was finished, the participants received the list of words used in the experimental blocks and were asked to indicate words whose meaning they did not know. If the participant did not know the meaning of a word, the trial containing that word was excluded from analysis. A total of 30 such trials (no more than four from a given participant) were excluded from the entire data set. The entire experiment lasted approximately 30 min.

### Results and discussion

**Fig. 1:**
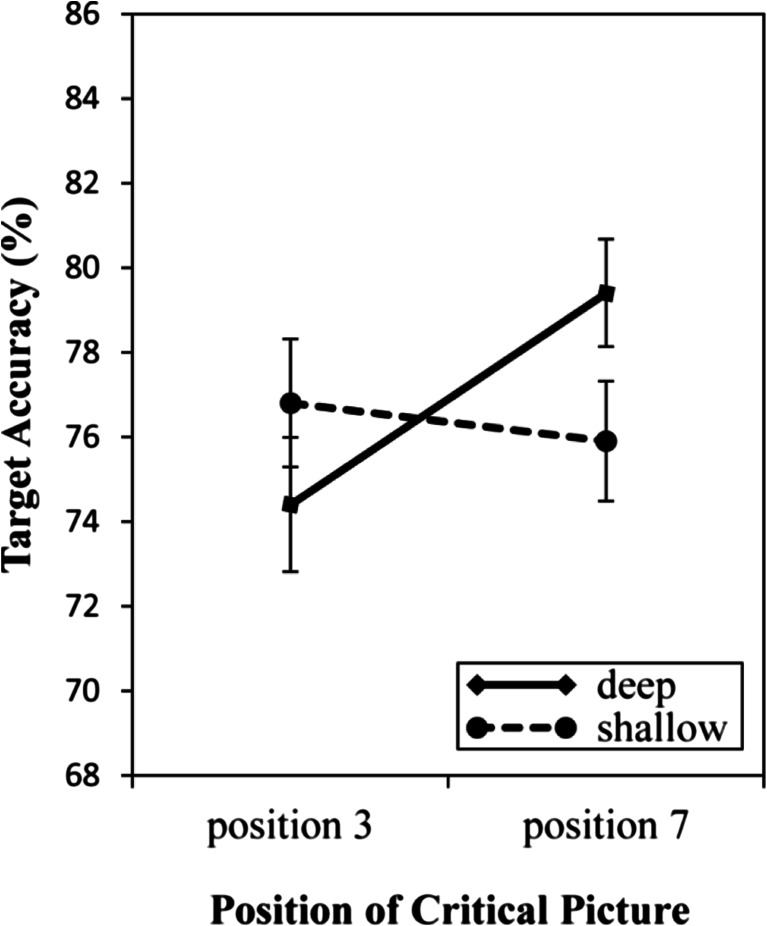
Experiment 1 mean accuracy in the digit identification task, as a function of encoding condition and Position of the Critical Picture. The digit target was presented in position 5. Error bars reflect standard errors of the mean

The average presentation duration of the digit was 43 ms. Animacy judgments in the deep processing condition were correct on 93.9% of the trials, and the mean reaction time (RT) was 1,088 ms. Case judgments in the shallow processing condition were correct on 97.7% of the trials, and had a mean RT of 809 ms. The differences in accuracy and RT between the two encoding conditions were significant, *t*(58) = 5.76, *p* < .001, *d* = 0.75, and *t*(58) = 13.81, *p* < .001, *d* = 1.74, respectively. The analysis of digit identification accuracy was restricted to trials in which the animacy or case judgment was correct. A 2 (position of critical picture: 3 or 7) $\times$ 2 (type of task: animacy or case judgment) $\times$ 2 (order: animacy-judgment or case-judgment trials first) repeated measures ANOVA was performed on mean accuracy in the digit identification task. The only effect to reach significance was the interaction of task and Position of the Critical Picture, *F*(1.28) = 5.17, *p* = .031, *μ*_*p*_^2^ = .16 (all other *F*s < 2.51, all other *p*s > .12). A follow-up, two-tailed pairwise *t* test showed significantly lower performance when the critical picture preceded the digit (74.4%) than when the critical picture was presented after (79.4%) the digit in the deep encoding condition, *t*(58) = 2.78, *p* = .007, *d* = 0.36, but not in the shallow-encoding condition (76.8% vs. 75.9%, *t*(58) = 0.44, *p* = .659; see Fig. 1). A regression analysis was conducted to determine if this capture effect, defined in terms of AB magnitude, depended on RT in the word processing task. The analysis revealed that RT on the animacy and case-judgment tasks was not a significant predictor of AB (β = .087, *p* = .348), accounting less than 1% of variance in the AB magnitude, *F*(1, 116) = 0.888, *p* = .348, *R* ^2^ = 0.008. Lastly, the results for the recognition memory task yielded *d’* = 1.24 for the shallow-encoding condition and *d’ =* 2.08 for the deep-encoding condition, reflecting a significant effect of depth of encoding, *t*(58) = 10.62, *p* < .001, *d* = 1.3, on recognition memory.

Taken together, the results of Experiment 1 show that pictures of words that had undergone deep encoding attracted attention and thereby resulted in an attentional blink effect in a subsequent RSVP task. In contrast, we found no evidence of such an attentional capture effect for pictures that corresponded to shallowly encoded words. Accordingly, it can be concluded that deep encoding increases the likelihood that an object will capture attention in a subsequent task. What remains to be determined, however, is whether this attentional capture effect indeed arose due to residual WM activation of the words. The goal of Experiment 2 was to address this matter.

## Experiment 2

In Experiment 2, we examined whether the attentional capture effect observed in the deep encoding of Experiment 1 was driven by residual WM activation. To address this matter, we examined if the capture effect would be abolished if participants had to perform an additional, demanding WM task directly after judging the word. Specifically, we reasoned that if the capture effect stemmed from residual WM activation, then the requirement to encode a large amount of new information in WM should abolish this effect because it would erase the residual WM activation of the word’s meaning (see also, Zhang et al. 2011). To test this hypothesis, we replicated the deep encoding condition of Experiment 1 with or without the presence of a demanding WM task directly after judging the word.

### Method

#### Participants

Thirty-three students (19 females, *M* = 20.2 years, *SD* = 1.84) of the English-language psychology bachelor program at the University of Groningen participated in the experiment for partial course credit. All had normal or corrected to normal visual acuity. The study was approved by the ethics committee of the psychology department. Informed written consent was obtained.

#### Apparatus and stimuli

Apparatus and stimuli were similar to those of Experiment 1, with the exception that Experiment 2 included an additional WM task. The stimuli for this task were similar to the stimuli used by Zhang and colleagues (2011) and consisted of shapes that each measured approximately 1.7° $\times$ 1.7° in visual angle. Four of these shapes were displayed for the WM task on each trial, and these shapes were positioned at the corner of an imaginary rectangle measuring 5.7° of visual angle horizontally and 4.1° vertically. In the memory test a probe shape was presented in the center of the screen, and participants had to judge whether this shape was present or absent in the memory display. The shape was present in the display in 50% of the trials.

#### Procedure

The procedure was identical to that in Experiment 1, except for the following. In the experiment, trials differed in whether the animacy task was followed by the memory set of four shapes or not. In the condition with this memory task (WM load condition), the memory set was displayed for 3,000 ms directly after participants responded to the word. In the condition without this additional WM task (no WM load condition), the animacy task was followed by a 3,000-ms blank screen. In both conditions, the RSVP digit identification task was presented next. In the WM load condition, the response for the digit identification task was followed by a WM test in which a single shape appeared on the screen for which participants had to indicate whether present or absent in the memory set.

### Results and discussion

**Fig. 2:**
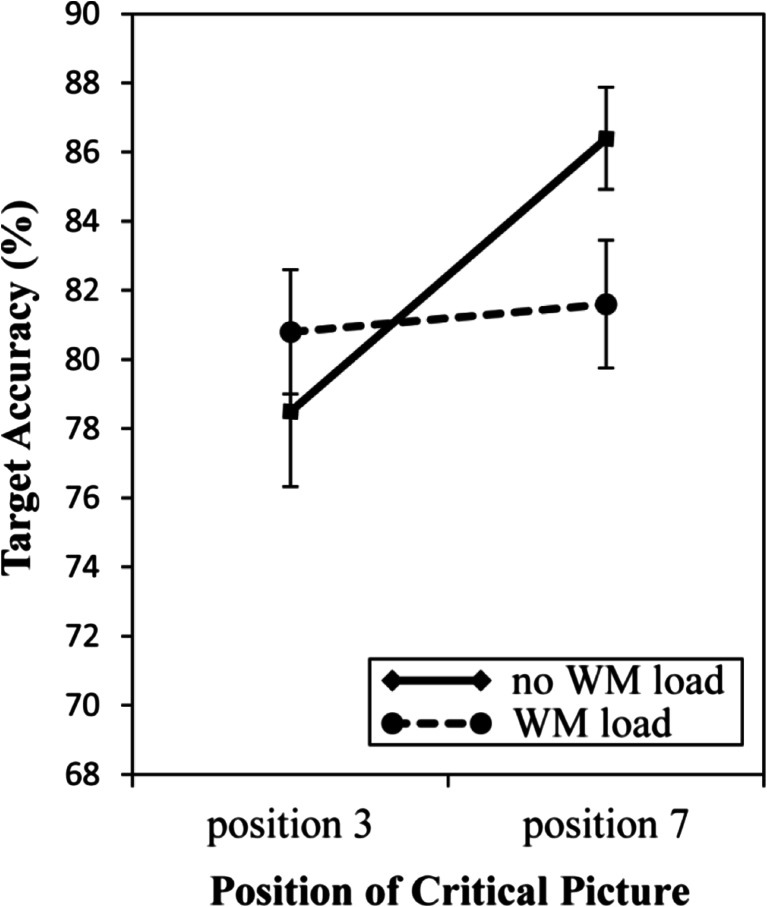
Results for Experiment 2. Mean accuracy in the digit-identification task, shown as a function of WM load condition and Position of Critical Picture. The target digit was presented in position 5. Error bars reflect standard errors of the mean

We excluded three participants that performed at chance level in the WM test. For the remaining 30 participants, mean accuracy in memory task was 82.3% correct. The average presentation duration of the digit was 50 ms. Animacy judgments were correct on 96.2% of the trials and had a mean RT of 1215 ms. The analysis of digit identification accuracy included only those trials in which the animacy-judgment task and the memory task—if present—were done correctly. A 2 (position of critical picture: 3 or 7) $\times$ 2 (WM load: load or no load) repeated measures ANOVA was performed on mean accuracy in the digit identification task. The main effect of Position of the Critical Picture was significant, *F*(1.29) = 5.67, *p* = .024, *μ*_*p*_^2^ = .16, with lower performance when the critical picture was presented before the digit (79.7%) than when the critical picture was presented after the digit (84.0%). There was no main effect of WM load, *F*(1.29) = 0.40, *p* = .536. Crucially, the interaction between Position of the Critical Picture and WM load was significant, *F*(1.29) = 5.07, *p* = .032, *μ*_*p*_^2^ = .15, indicating that performance was lower when the critical picture preceded the digit (78.5%) than when the critical picture was presented after (86.4%) the digit in the no WM load condition, *t*(29) = 3.01, *p* = .005, *d* = 0.64, but not in the WM load condition, 80.8% vs. 81.6%, *t*(29) = 0.37, *p* = .719. This pattern of results is illustrated in Fig. 2.

Taken together, the results of Experiment 2 replicate the finding of attentional capture by a picture that corresponded to a deeply encoded word, and they show that this capture effect was eliminated when encoding of the word was followed by a demanding memory task. This finding suggests that the attentional capture effect caused by deep encoding was indeed driven by residual WM activation of the encoded word.

## General discussion

In two experiments, the current study examined whether the likelihood of attentional capture by a picture depicting a previously processed word depends on whether this word had undergone shallow or deep encoding. In Experiment 1, we found that shallow encoding of a word did not result in attentional capture even though participants were found to perform well above chance in a recognition test for these words. In contrast, deep encoding did lead to attentional capture, and this effect was found to be abolished when participants were asked to perform a demanding WM task directly after the deep-encoding task.

Taken together, the results of the current study suggest that deep encoding of a word leads to memory activation, which in turns biases the allocation of attention towards matching stimuli in an unrelated, subsequent RSVP target identification task. A similar account was offered by Soto and Humphreys (2007), who found that verbalization of visual primes results in a memory-driven capture effect similar to that generated by items that are maintained in WM. In this study, participants were asked to verbalize a visual prime or read aloud a prime word, and after which they performed a visual search for a target. The results showed that search times were slower when one of the distractors shared a feature with the prime even though there was no instruction to remember the prime. Soto and Humphreys concluded that verbalization of the prime leads to WM activation of corresponding concept, which in turn biased attention in an unrelated subsequent task. Likewise, it can be argued that the animacy-judgment task used in the current study led to the activation of the corresponding object in WM, thus resulting in memory-driven attentional capture when a picture of the object was later encountered again in the RSVP task. Consistent with this account, the results of Experiment 2 showed that the capture effect was abolished by the requirement to perform an intermediate WM task, thus suggesting that the residual activation was overwritten by the requirement to fill WM with new information.

An additional important finding was that the results of the incidental memory test in Experiment 1 showed better performance in recognition of deeply rather than shallowly encoded words. Nevertheless, recognition of shallowly encoded words was also well above chance, suggesting that the meaning of these words must have been extracted, for else these words could not be recognized in a later memory test. The reason why deep and shallow encoding may lead to differences in attentional capture may be understood in view of the notion that WM activation may consist of different states (Nee & Jonides, 2011; Oberauer, 2002; Olivers et al., 2011). A particularly relevant account of WM activation can be found in Olivers et al., who distinguished between active and accessory WM representations. According to this account, the item relevant to the current task gains the status of a search template with a full access to sensory input. In contrast, items in WM that are not relevant to the task at hand, but which can still influence visual selection, receive a status of “accessory” item. Seen in light of this account, we propose that the semantic representations of deeply processed words in our task acted as strong accessory items that gained a representational state in WM that temporally guided the deployment of attention towards matching stimuli. Furthermore, we propose that the reason why shallow encoding did not lead to attentional capture is because the representations of these words were only weakly activated and therefore unable to guide attentional selection. Taken together, our findings can be taken as evidence for the relationship between strength of WM activation and the deployment of selective attention.

To conclude, our study shows that residual WM activation resulting from performing an animacy-judgment task is sufficient to guide attentional selection in a subsequent, unrelated RSVP task. Furthermore, we found that even though a case-judgment task also leads to the activation of semantic information, this activation was too weak to induce attentional capture. Our findings thus illustrate that the attentional capture paradigm developed by Pashler and Shiu offers an interesting means for studying residual WM activation. Future research will need to address whether the likelihood of attentional capture depends on whether information is held in WM actively or passively as a residue of earlier processing.

## Footnotes

1 An alternative explanation for this finding is that the capture effect is not due to guidance of attention by residual WM activation, but due to priming of word meaning. According to a priming account, any processing that activates an object’s representation should be sufficient to cause priming and thus lower the threshold for the subsequent identification of the object. Importantly, however, Soto et al. (2005) showed that the typical conditions for eliciting priming effects do not lead to attentional capture, thus arguing against a priming account of the findings of Pashler and Shiu (1999).

## References

[CR1] Awh E, Jonides J (2001). Overlapping mechanisms of attention and spatial working memory. Trends in Cognitive Sciences.

[CR2] Awh E, Vogel E, Oh SH (2006). Interactions between attention and working memory. Neuroscience.

[CR3] Baddeley A (2010). Working memory. Current Biology.

[CR4] Balota DA, Yap MJ, Cortese MJ, Hutchison KA, Kessler B, Loftis B, Treiman R (2007). The English Lexicon Project. Behavior Research Methods.

[CR5] Brysbaert M, New B (2009). Moving beyond Kučera and Francis: A critical evaluation of current word frequency norms and the introduction of a new and improved word frequency measure for American English. Behavior Research Methods.

[CR6] Carlisle NB, Arita JT, Pardo D, Woodman GF (2011). Attentional templates in visual working memory. Journal of Neuroscience.

[CR7] Cowan N (2005). Working memory capacity.

[CR8] Cowan N (2010). The magical mystery four: How is working memory capacity limited, and why?. Current Directions in Psychological Science.

[CR9] Craik FIM, Lockhart RS (1972). Levels of processing: A framework for memory research. Journal of Verbal Learning and Verbal Behavior.

[CR10] Craik FIM, Tulving E (1975). Depth of processing and retention of words in episodic memory. Journal of Experimental Psychology: General.

[CR11] Downing PE (2000). Interactions between visual working memory and selective attention. Psychological Science.

[CR12] Lund K, Burgess C (1996). Producing high-dimensional semantic spaces from lexical co-occurrence. Behavior Research Methods, Instruments, & Computers.

[CR13] Nee DE, Jonides J (2011). Dissociable contributions of prefrontal cortex and the hippocampus to short-term memory: Evidence for a 3-state model of memory. NeuroImage.

[CR14] Nieuwenstein MR, Van der Burg E, Theeuwes J, Wyble B, Potter MC (2009). Temporal constraints on conscious vision: On the ubiquitous nature of the attentional blink. Journal of Vision.

[CR15] Oberauer K (2002). Access to information in working memory: Exploring the focus of attention. Journal of Experimental Psychology: Learning, Memory and Cognition.

[CR16] Olivers CNL, Peters J, Houtkamp R, Roelfsema PR (2011). Different states in visual working memory: When it guides attention and when it does not. Trends in Cognitive Sciences.

[CR17] Paivio A, Yuille JC, Madigan SA (1968). Concreteness, imagery, and meaningfulness values for 925 nouns. Journal of Experimental Psychology.

[CR18] Pashler H, Shiu LP (1999). Do images involuntarily trigger search? A test of Pillsbury’s hypothesis. Psychonomic Bulletin & Review.

[CR19] Pillsbury, W. B. (1908). *Attention*. New York, NY: Macmillan.

[CR20] Raymond JE, Shapiro KL, Arnell KM (1992). Temporary suppression of visual processing in an RSVP task: An attentional blink?. Journal of Experimental Psychology: Human Perception and Performance.

[CR21] Schneider W, Eschmann A, Zuccolotto A (2002). E-Prime user’s guide.

[CR22] Snodgrass JG, Vanderwart M (1980). A standardized set of 260 pictures: Norms for name agreement, familiarity and visual complexity. Journal of Experimental Psychology: Human Learning & Memory.

[CR23] Soto D, Humphreys GW (2007). Automatic guidance of visual attention from verbal working memory. Journal of Experimental Psychology: Human Perception and Performance.

[CR24] Soto D, Heinke D, Humphreys GW, Blanco MJ (2005). Early, involuntary top-down guidance of attention from working memory. Journal of Experimental Psychology: Human Perception and Performance.

[CR25] Soto D, Humphreys GW, Heinke D (2006). Working memory can guide pop-out search. Vision Research.

[CR26] Szekely A, D’Amico S, Devescovi A, Federmeier K, Herron D, Iyer G, Bates E (2005). Timed action and object naming. Cortex.

[CR27] Vogel EK, McCollough AW, Machizawa MG (2005). Neural measures reveal individual differences in controlling access to working memory. Nature.

[CR28] Zhang B, Zhang JX, Huang S, Kong L, Wang S (2011). Effects of load on the guidance of visual attention from working memory. Vision Research.

